# Anti-Obesity Potential of Modified Pomelo-Peel Dietary Fiber-Based Pickering Emulsion

**DOI:** 10.3390/nu17193036

**Published:** 2025-09-23

**Authors:** Kaitao Peng, Shiyi Tian, Shuang Bi, Xian Cui, Kaili Gao, Yuhuan Liu

**Affiliations:** 1 School of Food and Health, Beijing Technology & Business University, Beijing 100048, China; pkt1811826@163.com (K.P.); bishuang@btbu.edu.cn (S.B.); 2 State Key Laboratory of Food Science and Resources, Engineering Research Center for Biomass Conversion, Ministry of Education, Nanchang University, Nanchang 330047, China; cuixian@ncu.edu.cn; 3 College of Animal Science and Technology, Jiangxi Agricultural University, Nanchang 330045, China; stian11@jxau.edu.cn; 4 College of Biological and Environmental Engineering, Jingdezhen University, Jingdezhen 334000, China; 5 Chongqing Research Institute of Nanchang University, Chongqing 402660, China

**Keywords:** insoluble dietary fiber emulsion, agricultural by-products, obesity, gut microbiota, short-chain fatty acids

## Abstract

Objectives: In response to the high prevalence of global obesity and associated metabolic diseases, this study aimed to investigate the effects of Pickering emulsions stabilized by cellulase-hydrolyzed pomelo peel insoluble dietary fiber (IDF), namely EPI and its octenyl succinic anhydride (OSA)-modified form (OSA-EPI), on alleviating high-fat diet (HFD)-induced metabolic disorders in mice. Methods: Male C57BL/6J mice were subjected to an HFD-induced obesity model. Biochemical index determination, histopathological examination, gut microbiota analysis, and short-chain fatty acids (SCFAs) analysis were used to study the potential efficacy of pomelo peel IDF-based emulsion (EPI and OSA-EPI) in alleviating obesity and related metabolic diseases. Results: The findings demonstrated that both emulsions effectively mitigated HFD-induced health impairments: reduced weight gain, improved blood glucose and lipid profiles, attenuated tissue steatosis and inflammation, and lowered oxidative stress. Furthermore, both EPI and OSA-EPI restored gut microbiota diversity, promoted the proliferation of beneficial bacterial taxa (e.g., *Akkermansia*), and inhibited the growth of harmful genera (e.g., *Muribaculum*, *Faecalibaculum*). These changes were accompanied by increased production of SCFAs. Conclusions: This study confirms that modified pomelo peel IDF can effectively exert the health intervention effect of IDF on obesity when used as an emulsion stabilizer, providing a robust scientific foundation for the application of emulsified dietary fibers in combating obesity and related metabolic disorders.

## 1. Introduction

With the escalating tempo of modern life, the high-fat dietary pattern is growing increasingly prevalent among the population, leading to a yearly escalation in the incidence of obesity and associated metabolic disorders [1]. Obesity, a major global health challenge, significantly increases the risk of chronic diseases such as cardiovascular disorders and type 2 diabetes [2,3,4]. High-fat diet (HFD)-induced obesity and metabolic syndrome are strongly associated with disruptions in gut microbiota composition [5]. Dietary modulation of the gut microbiota has thus gained attention as a promising strategy to mitigate obesity and its metabolic complications, driving ongoing research in nutritional and food science [6].

Dietary fiber (DF) plays a vital role in maintaining intestinal health and regulating body weight, blood lipids, and blood glucose levels [7]. As an essential component of DF, the functional scope of insoluble dietary fiber (IDF) has expanded from traditional physical effects—such as increasing fecal bulk—to broader physiological regulatory functions, including modulating intestinal microecology, host immunity, and metabolic processes [8,9]. Ge et al. found that bamboo-derived IDF can significantly promote the production of short-chain fatty acids (SCFAs), reduce the Firmicutes-to-Bacteroidetes (F/B) ratio, and improve the overall structure of the gut microbiota [10]. Importantly, IDFs sourced from different plants have shown promising potential in addressing health issues associated with HFD, such as obesity [11,12,13].

Previous studies conducted by our research group have demonstrated that IDF derived from pomelo peel, modified through cellulase hydrolysis, can serve as an effective Pickering emulsion stabilizer [14]. Further grafting modification with octenyl succinic anhydride (OSA) significantly enhances its emulsifying properties, including improved in vitro digestion characteristics of lipids and enhanced antioxidant capacity [15]. However, when pomelo peel-derived IDF is utilized as a stabilizer in Pickering emulsions, a critical question is whether the dense physical barrier formed at the oil–water interface might excessively restrict interactions between the IDF and the intestinal microbiota or host tissues, thereby impairing its bioactive functions—including microbiota modulation and anti-obesity effects. Moreover, it is unclear if this stability-enhancing barrier also delays lipid digestion and absorption to an undue extent. Such a delay could lead to excessive undigested lipids reaching the distal large intestine, potentially worsening HFD-induced metabolic disorders such as obesity and counteracting the beneficial effects of IDF. To the best of our knowledge, this issue has received little attention to date.

Therefore, this study selected two types of emulsions—EPI (prepared using cellulase-hydrolyzed pomelo peel IDF) and OSA-EPI (modified by OSA grafting on cellulase-hydrolyzed pomelo peel IDF), to systematically evaluate their roles and potential limitations in health regulation. We propose a central hypothesis: the physical barrier formed at the interface of such emulsions can modulate lipid digestion while preserving the prebiotic activity of IDF. To test this hypothesis, a high-fat diet (HFD)-induced obese mouse model was established, and a comprehensive assessment was conducted across multiple parameters, including weight changes, blood glucose and lipid levels, major organ status, expression of inflammatory factors, oxidative stress response, and composition of gut microbiota. This article not only delves deeply for the first time into the possible “functional barrier effect” and “risk of digestion delay” of pomelo peel IDF-based emulsion in the actual physiological environment of the body, but also provides a scientific basis for the potential efficacy of this type of emulsion in alleviating obesity and related metabolic diseases, thereby laying a theoretical foundation and providing technical references for the development of a new generation of functional dietary fiber foods. It helps promote the development of healthy dietary choices for the public.

## 2. Materials and Methods

### 2.1. Materials and Reagents

Majia pomelos (*Citrus. grandis* (L.) Osbeck) were purchased from Shangrao, Jiangxi Province. Cellulase (derived from Aspergillus Niger) was purchased from Solarbio Biotechnology Co., Ltd. (Beijing, China). OSA was derived from Nanjing Gu-Tian Chemical Co. (Nanjing, China). Animal feed is provided by Xiaoshu Youtai Biotechnology Co., Ltd. (Beijing, China) and includes standard and high-fat model mouse food. All other chemicals and reagents required for the experiment were purchased from Macklin Biochemical Technology Co., Ltd. (Shanghai, China), and analytical or higher-quality reagents were used throughout the experiment.

### 2.2. Emulsion Preparation

EPI and OSA-EPI were prepared according to the method we previously reported [14,15], and further detailed in Text S1.

### 2.3. Material Characterization

The particle size distribution, D(4,3), and Span value of the emulsions were analyzed using a Mastersizer 3000 particle size analyzer (Malvern Instruments, Malvern, UK). The microstructure and droplet aggregation of the emulsions were characterized by laser confocal microscopy (CLSM) (Carl Zeiss, Jena, Germany). The apparent viscosity and viscoelastic modulus (G′/G″) of EPI and OSA-EPI were evaluated using a rotational rheometer (Anton Paar, Graz, Austria) [16]. The ζ-potentials of the emulsions were measured using a zeta potentiometer (Malvern Instruments, Malvern, UK).

### 2.4. Animals and Experimental Design

**Figure 1:**
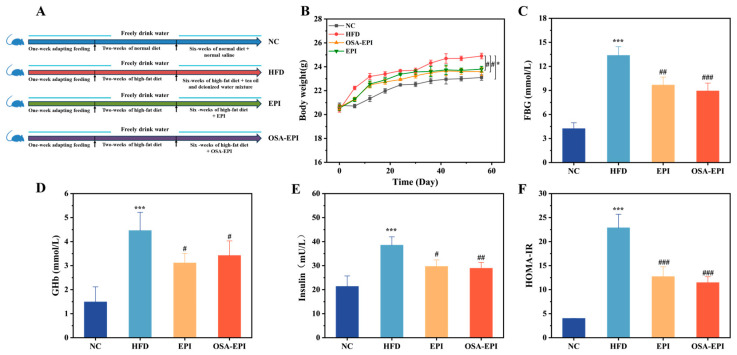
(**A**) Feeding pattern of animal experiment. Effects of emulsion intervention on (**B**) body weight, (**C**) FBG content, (**D**) GHb content, (**E**) Insulin value, and (**F**) HOMA-IR index in HFD mice. Note: “*” in (**B**–**F**) represents a significant difference compared with group NC, where * indicates *p* < 0.05, *** indicates *p* < 0.001; “#” represents a significant difference compared with group HFD, where # indicates *p* < 0.05, ## indicates *p* < 0.01, and ### indicates *p* < 0.001.

Thirty-two SPF-grade male C57BL/6J mice (6 weeks old, weighing 20 g ± 2 g) were procured from SPF Biotechnology Co., Ltd. (Beijing, China). The study was approved by the Experimental Animal Welfare Ethics Review Committee of Nanchang University (No.: SYXK(Gan)2021-0004) and adhered to the guidelines for the care and use of laboratory animals. Following a one-week adaptation period under standard environmental conditions (temperature: 22–24 °C, humidity: 40–55%, 12 h light/dark cycle), the mice were randomly allocated into four groups (*n* = 8, divided into two cages with 4 mice per cage) [17,18,19]: normal control (NC) group (fed with standard feed, normal saline administered via gavage from the 3rd to the 8th week, 0.75 mL per dose × 3 doses daily); HFD group (fed with HFD and gavaged with tea oil–water mixture following the same protocol, oil-to-water ratio was 2:8); EPI group (fed with HFD and gavaged with EPI following the same protocol); OSA-EPI group (fed with HFD and gavaged with OSA-EPI following the same protocol). It should be particularly noted that the oil–water mixture administered by gavage in the HFD group was an unhomogenized physical mixing system. Its physical properties included thermodynamic instability, rapid phase separation after standing still, the oil phase was dispersed in the form of large droplets visible to the naked eye, and it lacked a stable interface structure, which led to the rapid and complete digestion and absorption of lipids in the digestive tract. In contrast, EPI and OSA-EPI are Pickering emulsions stabilized by IDF from pomelo peel (PIDF/OSA-PIDF), with uniform droplet distribution and particle size at the micrometer level (specific characterization data are shown in Table S1). A dense solid barrier is formed at the interface, endowing the emulsion with excellent kinetic stability. And it can effectively delay the enzymatic hydrolysis and absorption process of lipids in the intestinal tract. In the EPI and OSA-EPI groups, the daily gavage dose of PIDF and OSA-PIDF was 844 mg per kg of body weight (calculated based on an average body weight of 20 g for mice). The experimental design is illustrated in Figure 1A. Throughout the experiment, body weight was recorded every 7 days. Prior to the termination of the experiment, all mice were subjected to a 12 h fasting period, followed by aseptic collection of fecal samples. Subsequently, the mice were euthanized under anesthesia, and serum and tissue samples were harvested. All samples were rapidly frozen in liquid nitrogen and stored at −80 °C for further analysis. There were no criteria set for including and excluding animals during the experiment. In addition, the group sequence of daily intragastric administration was randomized by the random number table method to eliminate the possible systematic error introduced by the operation time sequence.

### 2.5. Blood Glucose Index Analysis

At the end of the experiment, fasting blood glucose (FBG) of the mice was measured after 12 h of fasting (tail vein blood collection method). The blood was collected by eyeball extraction and centrifuged at 4 °C (5000× *g*, 15 min) after the anticoagulation centrifuge tube. The upper serum was collected and stored in the refrigerator at −80 °C for future use. The content of glycosylated hemoglobin (GHb) in mice was determined with a glycosylated hemoglobin analyzer and kit. Serum insulin levels were measured by an ELISA kit purchased from Nanjing Jiancheng Bioengineering Institute (Nanjing, China). In addition, the insulin resistance index (HOMA-IR) was calculated according to the following formula:

$$\mathrm{HOMA}\text{-}\mathrm{IR}=\mathrm{Insulin}\;\mathrm{level}\left(\mu U/\mathrm{mL}\right)\times \mathrm{FBG}(\mathrm{mmol}/L)/22.5$$

### 2.6. Detection of Biochemical Indexes

The total cholesterol (TC), triglyceride (TG), high-density lipoprotein cholesterol (HDL-C), and low-density lipoprotein cholesterol (LDL-C) levels were measured using a fully automated biochemistry analyzer (Agilent, Santa Clara, CA, USA). The interleukin-1β (IL-1β), interleukin-10 (IL-10), interleukin-6 (IL-6), and tumor necrosis factor-α (TNF-α) levels in the serum were analyzed using Mouse ELISA Kits. According to the superoxide dismutase (SOD), catalase (CAT), and malondialdehyde (MDA) kits, the frozen serum samples of mice were thawed and diluted to an appropriate concentration. The antioxidant properties of the serum of mice in different groups were determined by referring to the steps in the instructions. All these kits were purchased from Nanjing Jiancheng Bioengineering Institute (Nanjing, China).

### 2.7. Histological Analysis

The steps for oil red O staining of carotid tissue are as follows: After sampling, the intact carotid tissue was immersed in 10% neutral buffered formalin, and post-fixation was performed at 4 °C for 48 h. The fixed tissues were thoroughly rinsed with phosphate-buffered saline (PBS) and then successively placed in gradient sucrose solutions (10%, 20%, and 30% *w*/*v* in PBS or deionized water) and dehydrated at 4 °C until the tissues sank to the bottom. The surface liquid of the dehydrated tissue was absorbed by filter paper and placed in the optimal cutting temperature compound, then rapidly frozen in dry ice. Subsequently, the cryostat was used for continuous sectioning (with a thickness of 5–10 μm) and attached to the slide. After the sections were balanced at room temperature, they were immersed in 60% isopropanol for 5 to 10 min to promote the binding of lipids and dyes. Pour out the isopropyl alcohol and immediately add the freshly prepared and filtered Oil Red O working solution. Immerse for 10–15 min in the dark at room temperature or 37 °C. Take out the sections and rapidly differentiate them in 60% isopropyl alcohol for several seconds (until the background is colorless), then rinse with distilled water or PBS gently. Finally, after sealing with a water-based mounting agent, the images were immediately observed and collected under the Light Microscope (Nikon, Tokyo, Japan).

Hematoxylin and eosin (H&E) staining procedures are as follows: formalin-fixed tissue (liver, spleen, and kidney) is embedded in paraffin wax, and slices of 8 μm thickness are cut at a certain distance and placed on slides, then H&E staining is performed, and images are observed under a microscope.

A semi-quantitative scoring system was used to evaluate the pathological changes in Steatosis grade, Lobular inflammation, and Ballooning in the liver. The degree of lesions in the germinal center or white medulla of the spleen, the degree of glomerular cell proliferation, the degree of quantity reduction, and the degree of renal tubular dilation were evaluated. All histological scores and measurements were conducted independently by at least two researchers (blinded investigators) who were unaware of the groupings to ensure the objectivity of the assessment.

### 2.8. Determination of SCFAs

The content of SCFAs in mouse feces was determined by referring to the experimental method of Hu et al. and analyzed by gas chromatography (Agilent, Santa Clara, CA, USA) [20]. The sample was thawed at −80 °C and added with deionized water, mixed well, shaken, ultrasound for 5 min, ice bath for 2 h, centrifuged, and the supernatant collected then, a 0.22 µm water filter membrane was prepared for machine analysis. The chromatographic conditions were as follows: HP-FFAP column; The initial temperature of the column box was 75 °C, and the holding time was 1 min. Temperature rise condition 20 °C/min to 180 °C, 40 °C to 220 °C; The feed temperature of the flame ionization detector was 250 °C, the loading amount was 1 µL, the shitter ratio was 5:1, and the flow rate of nitrogen was 3.0 mL/min. The flow rates of hydrogen and air are 40 mL/min and 400 mL/min, respectively.

### 2.9. Gut Microbiota Analysis

After the experiment, fecal samples of mice were collected and rapidly frozen in liquid nitrogen, and then stored in an ultra-low temperature environment of −80 °C for analysis. The subsequent 16S rRNA gene sequencing and bioinformatics analysis were entrusted to Shanghai Majorbio Biotechnology Co., Ltd. (Shanghai, China) for completion.

The specific process is as follows: The total genomic DNA of the fecal microbiome is extracted using the QIAamp^®^ Fast DNA Stool Mini Kit (QIAGEN N.V, Hilden, Germany). The quality of DNA extraction was preliminarily evaluated by 1% agarose gel electrophoresis and quantified using a UV spectrophotometer. The V3-V4 hypervariable region of the 16S rRNA gene (338F: ACTCCTACGGGAGGCAGCA; 806R: GGACTACHVGGTWTCTAAT) was selected for PCR amplification. After the amplification products were detected by 2% agarose gel electrophoresis, they were precisely quantified using the QuantiFluor™-ST Blue fluorescence quantification system (Promega, Madison, WI, USA) and then mixed in equal molar ratios to construct the Illumina sequencing library. Library construction includes index sequence ligation, gel recovery and purification of PCR products, elution with Tris-HCl buffer, and denaturation with sodium hydroxide to generate single-stranded DNA fragments. Subsequently, paired-end sequencing was performed on the Illumina NovaSeq platform. The sequencing process includes bridge PCR amplification to generate clusters, sequencing while synthesizing (SBS), and fluorescence signal acquisition.

The PE reads obtained by Illumina sequencing were first spliced according to the overlapping relationship. Meanwhile, the quality of the sequences was controlled and filtered, and the samples were distinguished. Then, OTU cluster analysis and species classification analysis were conducted.

### 2.10. Statistical Analysis

The data results are represented by the mean plus or minus the standard deviation (mean ± SD). All other biochemical indices, histological, and morphological analyses in this study were performed using samples from all mice in each group (*n* = 8), except gut microbiota analysis, which excluded 3 samples (final *n* = 5) due to poor sequencing quality of some samples. Statistical analysis was performed using SPSS 19.0 software, and One-way ANOVA was used to analyze the significant differences between the data. Duncan’s test was used to check for homogeneity of variances. The confidence level was set at two-tailed 95%, i.e., *p* < 0.05. The drawing was performed using Origin 2024 software. Sequencing data analysis was completed on the Majorbio cloud platform (www.majorbio.com, accessed on 5 September 2025). β diversity analysis was conducted using the Bray–Curtis distance for principal coordinate analysis (PCoA). For the analysis of different-species, LEfSe (linear discriminant analysis effect size) was used for inter-group comparison: Firstly, non-parametric Kruskal–Wallis (KW) and rank tests were used to test the species abundance differences between populations to obtain species with significant differences; Then, the Wilcoxon rank sum test was used to examine the consistency of the differences among different species in subgroups among different groups. Finally, linear discriminant analysis (LDA) was adopted to estimate the degree of influence of different species between groups.

## 3. Results

### 3.1. Physicochemical Properties and Stability Mechanism Basis of EPI and OSA-EPI

The fundamental physicochemical properties of EPI and OSA-EPI employed in this study have been thoroughly investigated and documented in prior research [14,15]. To validate the system’s stability and establish a reference for functional evaluation, we re-measured the particle size distribution, interfacial charge properties, microstructural characteristics, and rheological behavior of freshly prepared emulsions (Figure S1 and Table S1). As illustrated in Figure S1A,D and Table S1, OSA modification results in reduced droplet size, a more uniform size distribution (shifted toward smaller particles), and significantly improved physical stability—findings that align closely with previous reports [15]. Additionally, rheological analysis (Figure S1B,C) confirms the characteristic shear-thinning behavior, as well as higher viscosity and modulus values in OSA-EPI, indicating a stable gel-like network structure.

Regarding the core mechanisms underlying emulsion formation and stability, earlier studies have clearly demonstrated that EPI is essentially a composite system wherein PIDF tightly encapsulates oil droplets and forms a three-dimensional network. Its stability arises from a mechanism analogous to Pickering particle stabilization, combined with the reinforcing effect of the physical network [14]. More notably, OSA modification markedly enhances both the stability and functional potential of the emulsion. This enhancement primarily involves the formation of a dense hydrophobic interfacial barrier, reinforcement of the internal gel network, improvement in digestive resistance and freeze–thaw stability, and increased electrostatic repulsion that prevents droplet coalescence [15].

In summary, previous investigations have comprehensively elucidated the superior physicochemical attributes of EPI and OSA-EPI—including smaller droplet size, elevated viscosity, and stronger gel network structures—as well as their intrinsic stability mechanisms. Particularly, OSA modification contributes significantly to enhanced interfacial barrier effects and digestive resistance. The retested data from this study (Figure S1) further confirm the consistent stability of these emulsion systems, thereby providing a robust material basis for their subsequent application in HFD-induced obese mouse models and for in-depth exploration of their impact on obesity-related health indicators. Based on these findings, the following section focuses on the intervention effects of EPI and OSA-EPI intake in an obese mouse model.

### 3.2. Effect of EPI and OSA-EPI on Body Weight of HFD Mice

During the entire 8-week experimental period, the body weight of mice in all groups exhibited a consistent upward trend (Figure 1B). Compared with the NC group, the weight gain curve in the HFD group was significantly shifted upward (*p* < 0.05), indicating that the HFD induced a marked increase in body weight. Notably, the rate of weight gain in the emulsion intervention groups (EPI and OSA-EPI) was significantly slower than that in the HFD group (*p* < 0.05), with no statistically significant difference observed between the EPI and OSA-EPI groups. At the end of the experiment, the percentage increases in body weight relative to the initial values were 11.2%, 22.1%, 16.1%, and 15.2% for the NC, HFD, EPI, and OSA-EPI groups, respectively. Among these, the weight gain in the OSA-EPI group was slightly lower than that in the EPI group. This phenomenon may be attributed to the enhanced emulsion stability and interfacial layer density achieved through OSA modification, which potentially reduces the efficiency of oil digestion and absorption. To sum it up, a 6-week intervention with insoluble dietary fiber emulsion derived from pomelo peel effectively suppressed excessive weight gain induced by an HFD.

### 3.3. Effects of EPI and OSA-EPI on FBG and Insulin Resistance Indices in HFD Mice

The effects of emulsion intervention on blood glucose and insulin resistance in HFD-fed mice are illustrated in Figure 1C–F. FBG levels in the NC group remained within the normal range (4–6 mmol/L), whereas those in the HFD group significantly increased to 13.37 mmol/L (*p* < 0.001), indicating severe hyperglycemia induced by the HFD. Following emulsion intervention, FBG levels decreased to 9.67 mmol/L and 8.93 mmol/L in the EPI and OSA-EPI groups, respectively, both of which were significantly lower than those in the HFD group (*p* < 0.01 and *p* < 0.001), confirming the potential of pomelo peel fiber emulsion to ameliorate hyperglycemia.

GHb, a marker reflecting average blood glucose levels over 4 to 8 weeks, showed a positive correlation with the risk of diabetic complications [21]. Consistent with the trend of fasting blood glucose, GHb levels in the HFD group were significantly elevated compared with those in the NC group (*p* < 0.001), while a slight downward trend was observed in the fiber emulsion intervention groups.

Serum insulin analysis revealed that insulin levels in the EPI and OSA-EPI group decreased significantly (*p* < 0.05 and *p* < 0.01) after intervention with pomelo peel IDF emulsion, suggesting that the emulsion can effectively regulate insulin homeostasis.

### 3.4. Effects of EPI and OSA-EPI on Lipid Profiles in HFD Mice

**Figure 2:**
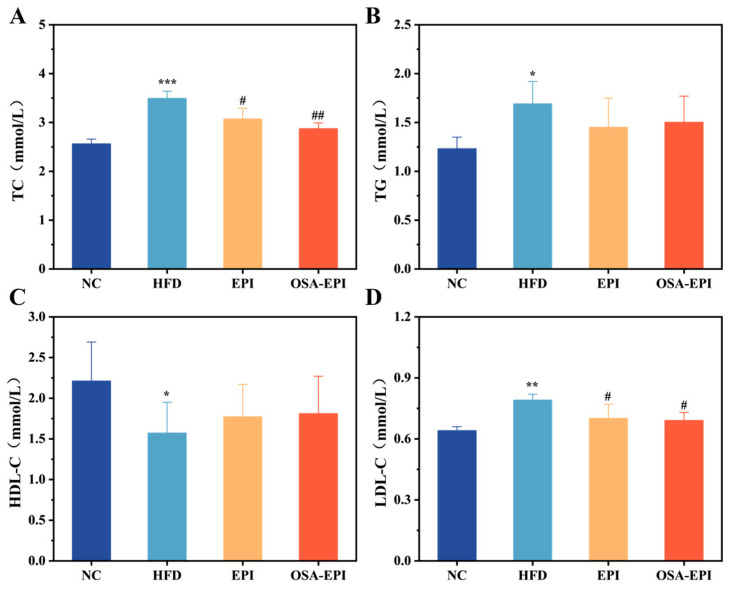
Effects of emulsions on serum (**A**) TC, (**B**) TG, (**C**) HDL-C, and (**D**) LDL-C levels in HFD mice. Note: “*” represents a significant difference compared with group NC, where * indicates *p* < 0.05, ** indicates *p* < 0.01, and *** indicates *p* < 0.001; “#” represents a significant difference compared with group HFD, where # indicates *p* < 0.05, and ## indicates *p* < 0.01.

Blood lipids can effectively reflect the degree of body obesity and health. The main mode of action of HDL-C in the body is to accelerate the movement of lipids from peripheral tissues to the liver for further metabolism, and high LDL-C is considered to be one of the indicators of cardiovascular disease [22]. As shown in Figure 2, compared with the NC group, the levels of TC (*p* < 0.001), TG (*p* < 0.05), and LDL-C (*p* < 0.05) in the serum of mice in the HFD group were significantly increased, while the levels of HDL-C were significantly decreased (*p* < 0.01). Therefore, the high-fat feeding mode has an adverse effect on the blood lipid levels of mice. After 6 weeks of intervention, serum levels of TC, TG, and LDL-C in the EPI group and the OSA-EPI group were decreased, while HDL-C level was increased. There was no significant difference between the EPI group and the OSA-EPI group, but the reduction in the degree of TC was higher in the OSA-EPI group, which may be due to the tight fiber coating of oil in the OSA-EPI group. The efficiency of oil absorption and digestion in the emulsion is reduced. These results indicate that the insoluble dietary fiber emulsion of pomelo peel can effectively regulate the serum lipid level of mice with a high-fat diet, and reduce the dyslipidemia caused by a long-term high-fat diet.

### 3.5. Effects of EPI and OSA-EPI on the Morphology of HFD Mice Organs

**Figure 3:**
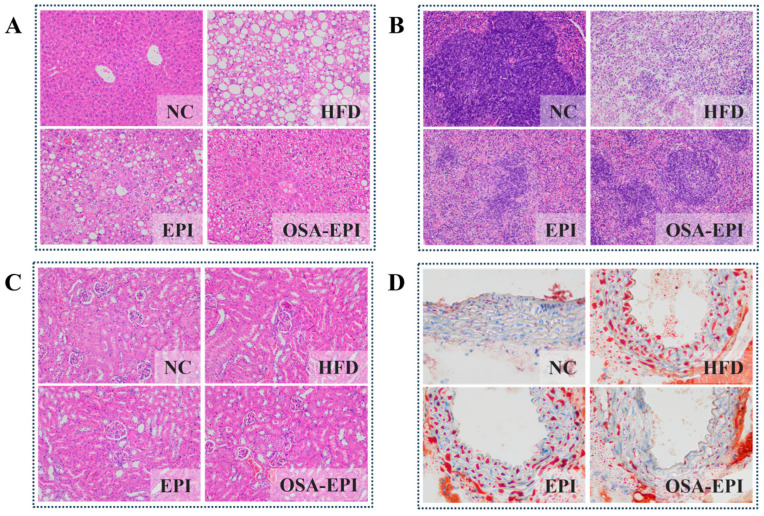
Effects of emulsion on the pathological analysis of (**A**) liver tissue, (**B**) spleen tissue, (**C**) kidney tissue, and (**D**) carotid tissue in HFD mice. Note: The scale bar was 100 μm. All image acquisition and evaluation were carried out in a blind state.

The probiotic effects of dietary fiber are often associated with reducing fat accumulation and inflammation in body organs such as the liver, subcutaneous tissue, muscle, and adipose tissue of the epididymis [23]. H&E staining results of liver, spleen, and kidney tissues in each group of mice are shown in Figure 3A–C. Compared with NC, after 8 weeks of HFD feeding, the liver structure of mice showed significant abnormalities, manifested as disordered arrangement of hepatocytes, increased intracellular lipid vacuolates, and a large number of macrophage infiltrations, with significant hepatic steatosis and inflammatory responses (Figure 3A). After intervention with EPI and OSA-EPI, the degree of liver lesions was reduced by 42.65% and 58.82%, respectively (Figure S2C). Consistent with this result, Figure S2A,B show that emulsion intervention can significantly reduce the contents of TC and TG in the liver.

In the spleen tissue, after HFD intervention, structural disorders of the medullary cords and medullary sinuses occurred, and cell necrosis and nuclear fragmentation could be observed. Compared with the HFD group, the degree of spleen structure damage in the EPI and OSA-EPI groups was reduced by 39.57% and 58.27%, respectively (Figure S2D), and the tissue structure tended to be regular (Figure 3B).

Similarly, after intervention with EPI and OSA-EPI, the phenomena of significant proliferation of glomerular endothelial cells, vasodilation and congestion, and inflammatory cell infiltration caused by HFD were significantly alleviated (Figure 3C).

Further oil red O staining results (Figure 3D) showed that in the HFD group of mice, a large number of large-volume oil red-colored lipid droplets could be seen beneath the vascular endothelium, and fat-cell aggregation could be observed in some vascular lumens, indicating a significant accumulation of lipid in the arterial intima. In contrast, in the EPI and OSA-EPI intervention groups, the degree of lipid deposition in the vascular wall was significantly reduced, and both the number and size of lipid droplets decreased.

The above results indicate that the Pickering emulsion constructed with modified pomelo peel IDF as the emulsifier can significantly alleviate the multi-organ tissue lesions and vascular lipid deposition induced by long-term high-fat diet without affecting the total energy intake. It is particularly worth noting that the OSA-EPI group demonstrated a better protective effect, with a milder degree of hepatic steatosis and a more complete spleen structure. This indicates that OSA modification may further delay the digestion and absorption process of lipids by enhancing the stability of the emulsion.

### 3.6. Effects of EPI and OSA-EPI on Inflammation and Oxidative Stress in HFD Mice

Obesity, as the central phenotype of metabolic syndrome, is frequently associated with chronic low-grade inflammation and oxidative stress imbalance [24]. Serum biochemical analysis (Table 1) revealed that the MDA content in the HFD group was significantly higher than that in the NC group (*p* < 0.05), indicating severe oxidative damage to the organism. In contrast, MDA levels were significantly reduced in the pomelo peel IDF emulsion intervention groups (EPI and OSA-EPI) compared to the HFD group (*p* < 0.05), confirming their efficacy in mitigating oxidative stress. Within the antioxidant enzyme system, SOD activity was markedly lower in the HFD group than in the NC group (*p* < 0.05). Emulsion intervention enhanced SOD activity to 1.35 times (EPI) and 1.29 times (OSA-EPI) that of the HFD group (*p* < 0.05). The trend in CAT activity paralleled that of SOD (HFD group < intervention group < NC group), indicating that emulsion intervention could synergically enhance antioxidant defense by catalyzing the clearance of superoxide anions and the decomposition of hydrogen peroxide (H_2_O_2_) [25,26].

Further analysis of inflammatory markers highlighted the pro-inflammatory state characteristic of the HFD group: Pro-inflammatory cytokines TNF-α, IL-1β, and IL-6 increased by 39.35%, 22.49%, and 60.85%, respectively, compared to the NC group (*p* < 0.05), while the anti-inflammatory cytokine IL-10 (which suppresses innate and adaptive immune inflammation) decreased by 28.26% (*p* < 0.05) [27]. Notably, IL-1β exacerbates inflammatory injury through the induction of ICAM-1 expression [28]. IDF emulsion intervention restored the balance of these inflammatory factors.

### 3.7. Effects of Insoluble Dietary Fiber Emulsion on Intestinal SCFAs in HFD Mice

**Figure 4:**
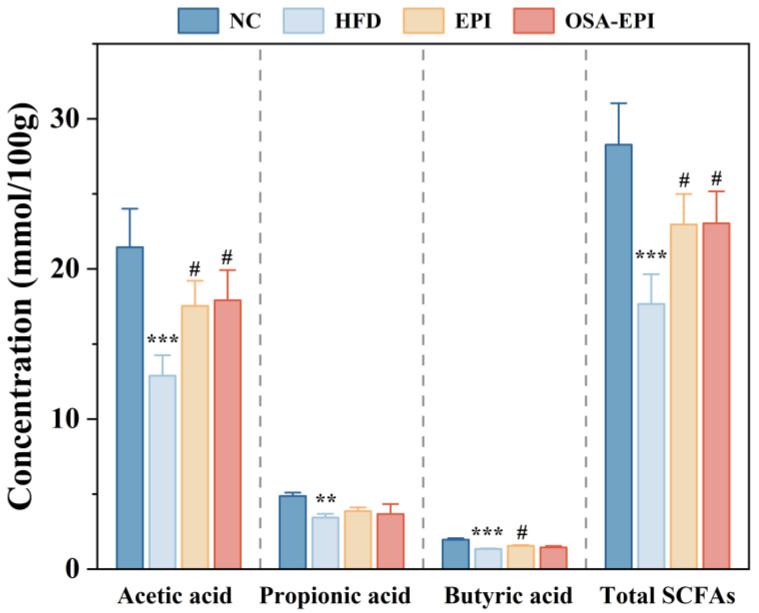
Effects of emulsion on the SCFA content in the colonic samples in obese mice. Note: “*” represents a significant difference compared with group NC, where ** indicates *p* < 0.01, and *** indicates *p* < 0.001; “#” represents a significant difference compared with group HFD, where # indicates *p* < 0.05.

SCFAs are the final products of indigestible polysaccharides fermented by specific intestinal anaerobic bacteria, which play an important role in host physiology and energy homeostasis, lipid synthesis, and immune regulation [28]. The effect of insoluble dietary emulsion intervention on SCFA content in the intestinal contents of mice with HFD is shown in Figure 4. The total SCFAs and the contents of acetic acid, propionic acid, and butyric acid in intestinal contents of the HFD group were significantly lower than those of the NC group, while the total SCFA content and the contents of acetic acid, propionic acid, and butyric acid in intestinal contents of mice could be partially restored by emulsion intervention, which verified the effect of insoluble dietary fiber emulsion intervention on intestinal health of mice.

### 3.8. Effects of Insoluble Dietary Fiber Emulsion on Intestinal Flora in HFD Mice

**Figure 5:**
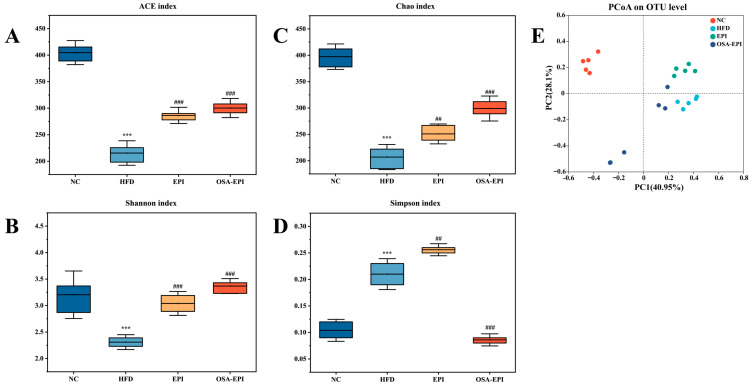
Effects of emulsions on gut microbial diversity of HFD mice. (**A**) ACE index; (**B**) Shannon index; (**C**) Chao index; (**D**) Simpson index; (**E**) Principal coordinates analysis (PcoA). Note: “*” represents a significant difference compared with group NC, where *** indicates *p* < 0.001; “#” represents a significant difference compared with group HFD, where ## indicates *p* < 0.01 and ### indicates *p* < 0.001.

To assess the impact of pomelo peel IDF emulsions on the intestinal microbiota of HFD mice, this study utilized fecal 16S rRNA high-throughput sequencing analysis. The results indicated that HFD severely impaired the α -diversity (richness and homogeneity) of the microbiota. Emulsion intervention effectively reversed this trend: the ACE index in the EPI group and the Chao and Shannon indices in the OSA-EPI group were significantly elevated compared with the HFD group (*p* < 0.05) (Figure 5A–D). PCoA further confirmed that fiber emulsion intervention can restructure the intestinal microbiota by restoring microbial diversity (Figure 5E).

**Figure 6:**
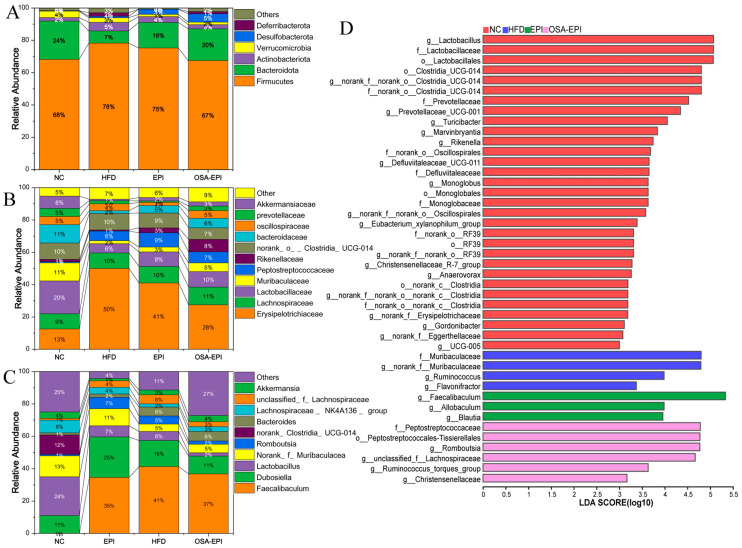
Gut microbial composition at the (**A**) phylum level, (**B**) family level, and (**C**) genus level. (**D**) The taxonomic species information with significant differences between groups was obtained by LEfSe analysis.

To further investigate the responses of specific micro-organisms, Figure 6A–C characterized the compositional changes in the intestinal microbiota of each experimental group at the phylum, family, and genus levels. Consistent with previous studies, Firmicutes, Bacteroidetes, and Actinobacteria constitute the predominant phyla of the intestinal microbiota in mice [29]. Compared to the NC group, the abundance of Firmicutes in the HFD group was significantly increased, while the abundance of Bacteroidetes was significantly decreased, leading to a marked elevation in the F/B ratio. This trend aligns with the typical characteristics observed in obesity models and corroborates findings from multiple studies reporting an increased F/B ratio in the gut flora of obese patients [30,31]. Notably, the F/B ratio in the emulsion intervention groups (EPI, OSA-EPI) was significantly reduced compared to the HFD group, suggesting that dietary fiber emulsion intervention may alleviate microbiota imbalances associated with HFD. Additionally, the abundances of two potentially harmful bacterial genera—Actinobacteriota and Deferribacterota—were significantly upregulated in the HFD group but were attenuated following emulsion intervention, further supporting the positive regulatory effects of dietary fiber emulsion on intestinal health.

At the family classification level (Figure 6B), the dominant intestinal flora primarily included Erysipelotrichaceae, Lachnospiraceae, Lactobacillaceae, Muribaculaceae, and Peptostreptococcaceae. Relative to the NC group, the relative abundance of Erysipelotrichaceae in the HFD group was significantly enhanced, a phenomenon previously associated with various metabolic disorders, including obesity in both humans and mice [32]. Emulsion intervention reduced the relative abundance of Erysipelotrichaceae, with a more pronounced effect observed in the OSA-EPI group. Furthermore, consistent with the findings of [33], the relative abundances of Lactobacillaceae, Muribaculaceae, and Akkermansiaceae were significantly diminished in the HFD group. Emulsion intervention effectively mitigated this downward trend. Given the critical roles of Lactobacillaceae and Muribaculaceae in intestinal energy metabolism and the regulation of blood glucose and lipids, maintaining their abundance is considered beneficial. These changes at the family level preliminarily indicate that pomelo peel fiber emulsion may influence obesity and related metabolic states in mice by modulating the abundance of obesity-associated microbiota.

Further analysis at the genus level revealed that the relative abundances of *Lactobacillus*, *Muribaculaceae*, and Clostridia-UCG-014 were significantly reduced in the HFD group, whereas the relative abundances of *Faecalibaculum*, *Dubosiella*, and *Romboutsia* were significantly elevated. Emulsion intervention alleviated these trends (Figure 6C). Multiple studies have demonstrated that the abundances of *Dubosiella* and *Romboutsia* are positively correlated with obesity [34,35]. The significant reduction in their abundances following emulsion intervention further validates its efficacy in combating obesity. Moreover, the abundance of *Akkermansia* in the EPI and OSA-EPI intervention groups was significantly higher than in the HFD group. As a mucin-degrading bacterium, *Akkermansia* is typically present at lower levels in pathological conditions such as alcoholic liver disease, hypertension, and obesity [36]. The intervention with IDF emulsion significantly increased the abundance of *Akkermansia*, suggesting it may reduce the risk of obesity and related metabolic diseases via this mechanism. This finding is consistent with research demonstrating the ability of insoluble dietary fiber to mitigate weight gain in mice fed with HFD [37].

The LEfSe results (Figure 6D) identified a total of 48 significantly different species across the phylum-to-genus levels. Distinctive species in the HFD group included Ruminococcus, *Flavonifractor*, and *Muribaculum*. Among these, Ruminococcus exhibits relatively high abundance in obese populations, is positively correlated with type II diabetes and non-alcoholic fatty liver disease, and has been identified as a potential trigger for Crohn’s disease [38]. In contrast, distinctive species in the EPI group included *Blautia*, *Faecalibaculum*, and *Allobaculum*. *Blautia* possesses probiotic properties, inhibiting inflammation and promoting SCFA production to maintain intestinal homeostasis. Its abundance is negatively correlated with obesity and type 2 diabetes, and dietary fiber supplementation can significantly enhance its abundance [39]. *Allobaculum*, an important SCFA-producing bacterium, is associated with a high-plasma HDL concentration, low circulating leptin concentration, and high-energy homeostasis gene expression [40]. It has also been reported to be significantly enriched in obesity models treated with bitter gourd powder, berberine, and raspberry polysaccharide [23,41,42]. Characteristic species in the OSA-EPI group included *Peptostreptococcals-Tissierellales*, *Romboutsia*, Peptostreptococcaceae, Christensenellaceae, and Lachnospiraceae. Among these, Christensenellaceae, belonging to Firmicutes, is regarded as a potential probiotic, with its abundance negatively correlated with body mass index (BMI), inflammation, and metabolic diseases [43].

**Figure 7:**
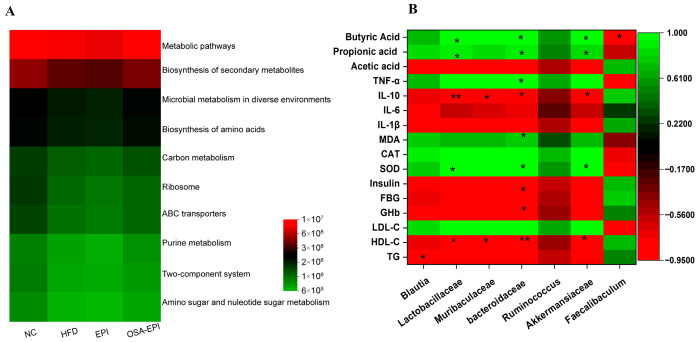
(**A**) Relative abundance distribution of metabolism function in gut microbiome based on KEGG; (**B**) The correlated heatmap with Spearman’s correlation coefficient between gut microbiota, and the biochemical parameters, * *p* < 0.05, ** *p* < 0.01.

The PICRUSt functional prediction analysis (Figure 7A) demonstrated that the microbiota functions in the intervention group were enriched in pathways such as KEGG metabolic pathways, secondary metabolite biosynthesis, environmental adaptive metabolism, amino acid biosynthesis, ribosome metabolism, and ABC transporter systems. Spearman correlation analysis (Figure 7B) further revealed that Lactobacillaceae, Bacteroidaceae, and Akkermansiaceae were significantly positively correlated with SCFAs production and SOD levels, while being significantly negatively correlated with anti-inflammatory cytokine IL-10 and HDL-C. Additionally, Muribaculum exhibited a negative correlation with IL-10 and HDL-C, whereas the abundance of Blautia was significantly correlated with total serum cholesterol.

## 4. Discussion

As a crucial component of DF, IDF enhances satiety and delays gastric emptying by absorbing water, swelling, and forming a gel-like substance in the stomach, thereby reducing food intake [44]. Additionally, IDF binds water molecules, increasing the volume and weight of fecal matter, accelerating intestinal transit, promoting defecation, and ultimately contributing to effective weight management [8]. The findings of this study demonstrate that when pomelo peel IDF was used as a Pickering emulsion stabilizer, it can still effectively play its role in slowing down the excessive weight gain in obese mice induced by HFD.

Due to its porous structure and large surface area, IDF has been shown to adsorb glucose and enzyme molecules, thereby inhibiting excessive glucose absorption and contributing to the regulation of blood glucose levels [45]. In this study, following intervention with EPI and OSA-EPI, HFD-induced hyperglycemia and glucose GHb levels were significantly reduced, accompanied by a decrease in serum insulin concentration. This result aligns with previous studies by Wang et al. and Yan et al., who, respectively, reported that kiwifruit-derived IDF and pumpkin-derived IDF effectively lower fasting blood glucose and insulin levels [46,47]. These findings further support the notion that pomelo peel IDF emulsion successfully retains the functional potential of IDF in reducing blood glucose and improving insulin resistance.

In terms of lipid metabolism regulation, functional groups present on the surface of DF possess the capacity to bind cholesterol and bile acids, thereby reducing dietary cholesterol absorption and promoting its excretion through feces, which helps prevent hyperlipidemia [48]. Research conducted by Rodriguez-Gutierrez et al. further supports the notion that IDF may exhibit strong cholesterol-binding properties [49]. As the core organ of lipid metabolism, the liver plays a crucial role in maintaining lipid homeostasis [50]. Dyslipidemia caused by HFD is often accompanied by lipid accumulation in the liver, which in turn induces liver steatosis, inflammation, and functional disorders, forming a vicious cycle [51]. Previous studies have shown that dietary fibers such as Hericium erinaceus IDF can significantly improve dyslipidemia and hepatic steatosis induced by HFD [52]. The results of this study demonstrate that pomelo peel IDF emulsions—both EPI and OSA-EPI—effectively retain the lipid-regulating function of IDF even when utilized as stabilizers in Pickering emulsions. Specifically, these emulsions significantly reduced serum levels of TC, TG, and LDL-C in HFD-fed mice. Furthermore, they markedly alleviated hepatic steatosis, inflammatory cell infiltration, and vascular lipid deposition. Notably, although OSA modification enhances emulsion stability, it does not induce excessive delays in lipid digestion or exacerbate metabolic disorders. Conversely, by delaying lipid absorption and enabling the orderly release and subsequent fermentation of DF, this system synergistically improves blood lipid profiles and the intestinal microenvironment.

HFD-induced obesity models are typically associated with intestinal microbiota dysbiosis, characterized by decreased microbial diversity, an elevated F/B ratio, and excessive growth of potentially pathogenic micro-organisms [5]. Studies have shown that intervention with pomelo peel IDF emulsion can effectively restore both α- and β-diversity of the gut microbiota and significantly reduce the F/B ratio. This observation aligns with multiple studies on DF modulation of the intestinal microbiota [37,47,52]. Notably, the emulsion intervention significantly promotes the proliferation of beneficial bacteria such as *Lactobacillus*, *Akkermansia*, and Christensenellaceae, which play crucial roles in maintaining intestinal barrier integrity, suppressing pathogenic bacterial growth, and modulating immune responses [43,53,54]. Simultaneously, the intervention inhibits the overgrowth of potentially harmful bacteria such as *Romboutsia*, which are known to produce pro-inflammatory and pro-oxidative metabolites that may exacerbate metabolic dysfunction [55].

The pomelo peel IDF emulsion modulates the composition of the gut microbiota and influences the production of microbial metabolites, thereby regulating host immune and inflammatory responses. As an emulsion stabilizer, pomelo peel IDF can reach the colon and be utilized by the intestinal flora, thereby generating beneficial metabolic products such as SCFAs, and ultimately activating and strengthening the intestinal barrier and multiple other mechanisms to alleviate systemic inflammation [8]. Meanwhile, the active substances encapsulated in the pomelo peel IDF emulsion may be metabolized by micro-organisms in the colon into more active compounds. These metabolites have significant antioxidant and anti-inflammatory activities, which can inhibit the activation of inflammatory pathways such as NF-κB and MAPK, reduce the expression of pro-inflammatory cytokines, and simultaneously upregulate the levels of anti-inflammatory cytokines [56].

Through these mechanisms, pomelo peel IDF emulsion shows multiple improvement effects on HFD-induced metabolic abnormalities, including significantly reducing weight gain, enhancing insulin sensitivity, improving dyslipidemia, modulation of gut microbiota and SCFA production, and alleviating inflammation and oxidative stress. OSA modification further enhanced the physiological protective effect of IDF by improving the stability of the emulsion, which was particularly evident in the OSA-EPI group.

Although this study provides valuable findings, some limitations still need to be recognized. Firstly, although an oil–water mixture was used as the blank emulsion control, its physical properties were different from those of the structured Pickering emulsion. In the future, a better-matching control group needs to be constructed to precisely analyze the IDF interface function. Secondly, the sample size for intestinal flora analysis is limited, and there are significant individual differences, which may affect the stability and universality of the results. In addition, although there is an association between changes in the microbiota and improvements in metabolic phenotypes, the key metabolites and causal mechanisms remain to be clarified. Finally, the research results, based on animal models, directly infer that humans need to be cautious. Future research should adopt multi-omics joint analysis, verify the causal relationship through antibiotic consumption and fecal microbiota transplantation experiments, and validate the mechanism and translational potential in models that are closer to human physiology.

## 5. Conclusions

In summary, the pomelo peel IDF emulsion, through its unique interfacial barrier structure, not only ensures the stability of the emulsion but also does not restrict the interaction between IDF and the intestinal microbiota or host tissues, nor does it overly delay the lipid digestion and absorption process, thereby avoiding the aggravation of metabolic disorders induced by HFD. This emulsion system can effectively utilize the multiple health benefits of IDF, significantly improving multiple indicators of obesity and related metabolic syndromes by regulating the composition of gut microbiota and the production of its metabolic products (such as short-chain fatty acids). These findings provide an experimental basis for the development of functional foods using agricultural by-products such as pomelo peel. Moreover, they highlight the translational potential of this Pickering emulsion system for human dietary interventions. For instance, it could be incorporated into functional food products—such as low-calorie dressings, healthy beverages, or meal-replacement formulations—as a fat replacer and prebiotic emulsion stabilizer. This offers a practical strategy for modulating postprandial lipid metabolism and gut health in humans, particularly in individuals with obesity or metabolic disorders.

## Figures and Tables

**Table 1:** Effects of emulsions on SOD, CAT, MDA, and inflammatory cytokine contents of HFD mice.

	NC	HFD	EPI	OSA-EPI
SOD (U/mL)	146.78 ± 8.98	97.21 ± 6.5 ***	112.34 ± 6.40 ^#^	119.6 ± 9.76 ^##^
CAT (U/mL)	18.89 ± 2.47	10.35 ± 1.53 **	16.64 ± 2.09 ^##^	17.03 ± 2.28 ^##^
MDA (nmol/mL)	4.63 ± 0.34	7.99 ± 0.57 ***	6.38 ± 0.62 ^##^	6.29 ± 0.59 ^##^
IL-1β (pg/mL)	127.54 ± 15.25	156.23 ± 9.78 *	132.77 ± 11.88 ^#^	129.12 ± 12.34 ^#^
IL-6 (pg/mL)	10.09 ± 2.12	16.23 ± 3.48 *	12.89 ± 2.34	13.78 ± 1.98
IL-10 (pg/mL)	7.67 ± 1.19	5.98 ± 0.98 *	6.37 ± 1.02	6.26 ± 1.21
TNF-α (pg/mL)	280.57 ± 19.34	390.98 ± 20.88 ***	338.76 ± 16.37 ^##^	340.12 ± 13.28 ^##^

Note: “*” represents a significant difference compared with group NC, where * indicates *p* < 0.05, ** indicates *p* < 0.01, and *** indicates *p* < 0.001; “#” represents a significant difference compared with group HFD, where # indicates *p* < 0.05 and ## indicates *p* < 0.01.

## Data Availability

The original contributions presented in the study are included in the article; further inquiries can be directed to the corresponding author. The 16S rRNA sequencing data presented in this study can be found in the NCBI Sequence Read Archive database, under accession number PRJNA1320701.

## Supplementary Materials

The following supporting information can be downloaded at: https://www.mdpi.com/article/10.3390/nu17193036/s1, Text S1. The preparation method of EPI and OSA-EPI. Table S1. D(4,3), zeta-potential and span of the freshly prepared emulsions. Figure S1. (A) Particle size distribution characteristics of EPI and OSA-EPI; (B) (C) Viscosity and modulus (G’, G”) of EPI and OSA-EPI. (D) Typical laser confocal spectra of EPI and OSA-EPI (scale bar: 50 µm). Figure S2. Effects of emulsion on the content of (A) liver TC, (B) liver TG, and the degree of lesions on (C) liver tissue, (D) spleen tissue, (E) kidney tissue, (F) carotid tissue in HFD mice. Figure S3: Representative original gas chromatograms (GC) of acetic acid, propionic acid, butyric acid standards and feces of mice in each group.
