# Supplementary material for: Anti-Obesity Potential of Modified Pomelo-Peel Dietary Fiber-Based Pickering Emulsion
Source: Nutrients. 2025 Sep 23;17(19):3036. doi: 10.3390/nu17193036 (PMC12526110; doi:10.3390/nu17193036)
Supplement: Supplementary file 1 [file nutrients-17-03036-s001.zip › nutrients-3866719 - supplementary.pdf]

Supplementary Materials

Anti-Obesity Potential of Modified Pomelo-Peel Dietary Fiber-Based Pickering Emulsion

Kaitao Peng [1,2,†], Shiyi Tian [3,†], Shuang Bi [1], Xian Cui [2], Kaili Gao [4,*] and Yuhuan Liu [2,5,*]

[1] School of Food and Health, Beijing Technology & Business University, Beijing, 100048, China; pkt1811826@163.com (K. P.); bishuang@btbu.edu.cn (S. B.)
[2] State Key Laboratory of Food Science and Resources, Engineering Research Center for Biomass Conversion, Ministry of Education, Nanchang University, Nanchang, Jiangxi, 330047, China; cuixian@ncu.edu.cn
[3] College of Animal Science and Technology, Jiangxi Agricultural University, Nanchang, 330045, China, stian11@jxau.edu.cn
[4] College of Biological and Environmental Engineering, Jingdezhen University, Jingdezhen, Jiangxi, 334000, China
[5] Chongqing Research Institute of Nanchang University, Chongqing, 402660, China
[*] Correspondence: gkaili189_2119@163.com (K.G.); liuyuhuan@ncu.edu.cn (Y.L.)
[†] These authors contributed equally to this work.

Contains

Number of pages in SM: 7

Number of texts in SM: 1

Number of tables in SM: 1

Number of figures in SM: 3

Text S1 The preparation method of EPI and OSA-EPI.

Specifically, take the pre-prepared IDF powder derived from pomelo peel and dissolve it in a phosphate buffer solution at pH 6.0. Add cellulase at a concentration of 200 U/g, incubate the mixture at 55°C for 2 hours, and subsequently inactivate the enzyme by boiling water bath treatment. Centrifuge the resultant mixture at 5,000×g for 15 minutes. Wash the precipitate twice with distilled water and freeze-dry it to obtain purified IDF (PIDF). Prepare a slurry using PIDF with a material-to-liquid ratio of 1:20 and adjust the pH to 8.5. Add a 5% (w/w) 2-octenyl succinic anhydride (OSA) solution at 85°C for 2 hours, followed by adjusting the pH to 7.0. Sequentially wash the precipitates with 90% ethanol and deionized water, and obtain OSA-modified PIDF (OSA-PIDF) via freeze-drying. Finally, mix 0.75% (w/w) PIDF or OSA-PIDF with tea oil (oil-to-water ratio was 2:8) in deionized water. Homogenize the mixture at 10,000 rpm for 2 minutes. Subsequently, perform emulsification using an ultrasonic cell disruptor (Ningbo Xinzhi Biotechnology Co., Ltd., Ningbo, China) at 20 kHz for 3 minutes (with a pulse cycle of 2 s on/2 s off). The resulting emulsions are labeled as EPI and OSA-EPI, respectively.

Table S1. D(4,3), zeta-potential and span of the freshly prepared emulsions.

	D4,3 (μm)	Zeta-potential (mV)	Span
EPI	22.30±2.21	-24.4±0.5	1.06±0.04
OSA-EPI	17.15±1.07	-42.3±0.5	0.93±0.12

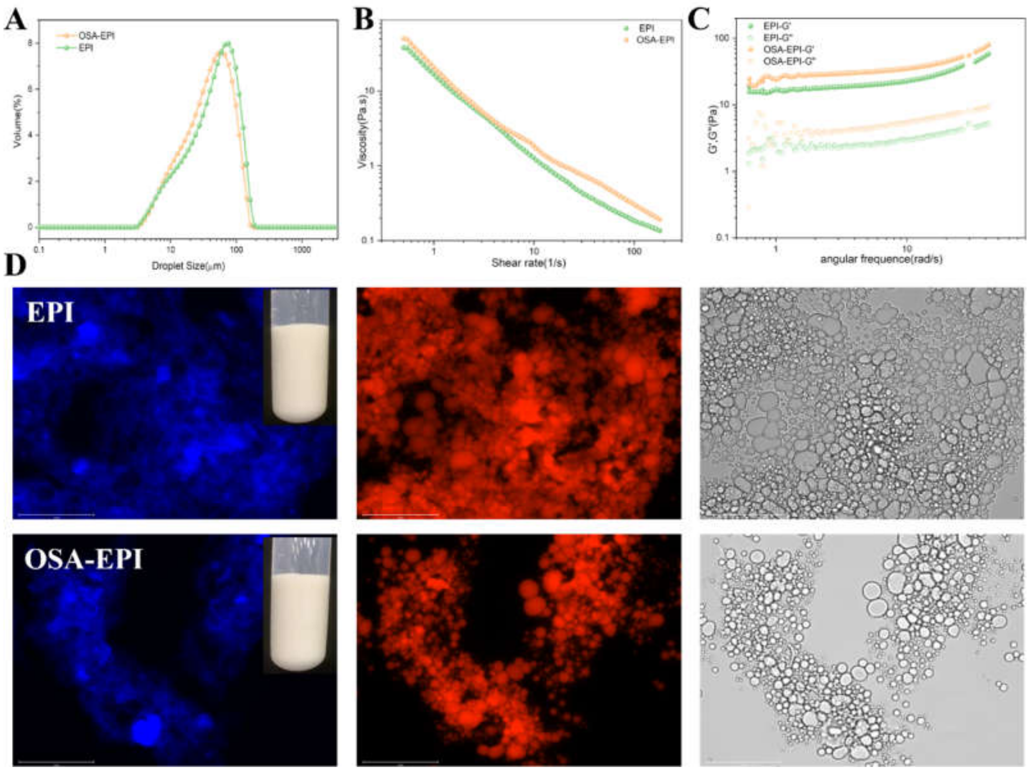

Figure S1. (A)Particle size distribution characteristics of EPI and OSA-EPI; (B)(C)Viscosity and modulus (G ', G") of EPI and OSA-EPI. (D) Typical laser confocal spectra of EPI and OSA-EPI (scale bar: 50 μm).

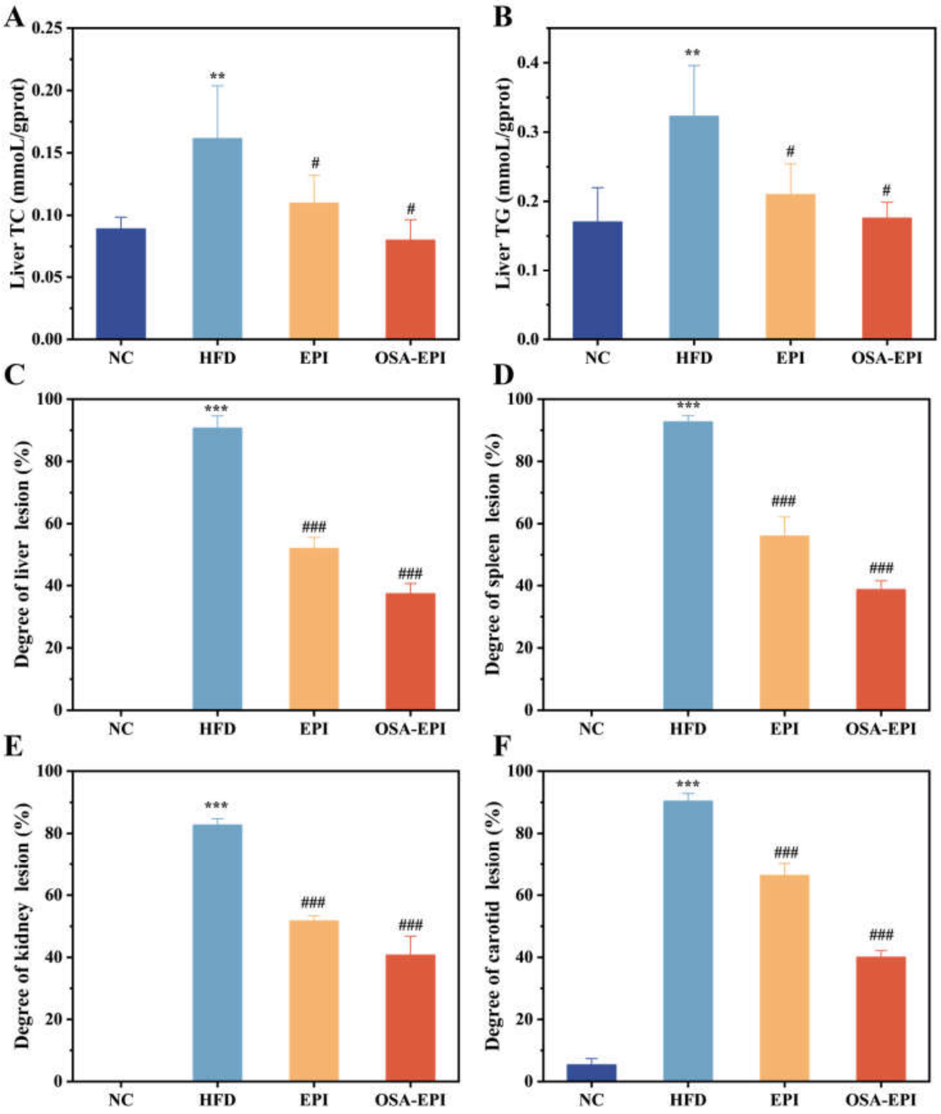

Figure S2. Effects of emulsion on the content of (A) liver TC, (B) liver TG, and the degree of lesions on (C) liver tissue, (D) spleen tissue, (E) kidney tissue, (F) carotid tissue in HFD mice. Note: "*" represents a significant difference compared with group NC, where ** indicates $P < 0.01$, *** indicates $P < 0.001$; "#" represents a significant difference compared with group HFD, where # indicates $P < 0.05$, ### indicates $P < 0.001$.

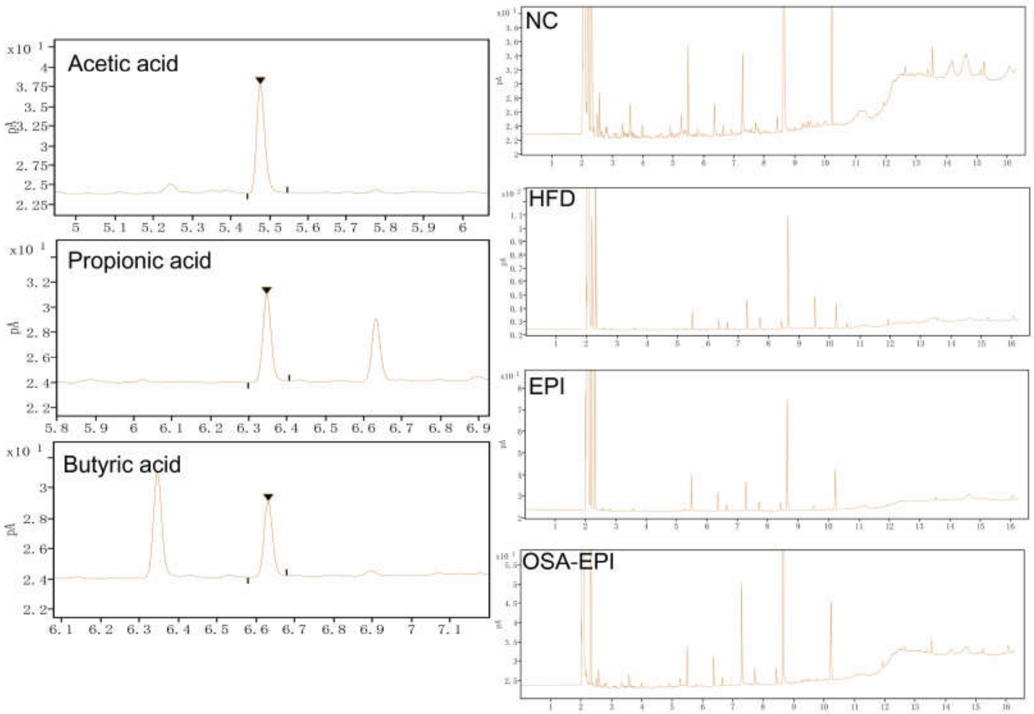

Figure S3. Representative original gas chromatograms (GC) of acetic acid, propionic acid, butyric acid standards and feces of mice in each group.
